# Safety and efficacy of ampreloxetine in symptomatic neurogenic orthostatic hypotension: a phase 2 trial

**DOI:** 10.1007/s10286-021-00827-0

**Published:** 2021-10-17

**Authors:** Horacio Kaufmann, Ross Vickery, Whedy Wang, Jitendra Kanodia, Cyndya A. Shibao, Lucy Norcliffe-Kaufmann, Brett Haumann, Italo Biaggioni

**Affiliations:** 1 Department of Neurology, Dysautonomia Center, NYU Langone Health, New York University School of Medicine, 530 First Avenue, Suite 9Q, New York, NY 10016 USA; 2 Theravance Biopharma Ireland Limited, Dublin, Ireland; 3 Formerly of Theravance Biopharma US, Inc., South San Francisco, CA USA; 4 Theravance Biopharma US, Inc., South San Francisco, CA USA; 5 Department of Medicine, Vanderbilt University Medical Center, Nashville, TN USA; 6 Formerly of Theravance Biopharma UK Limited, London, UK

**Keywords:** Ampreloxetine, Norepinephrine reuptake inhibitor (NRI), Neurogenic orthostatic hypotension (nOH), Synucleinopathies

## Abstract

**Purpose:**

In neurogenic orthostatic hypotension, blood pressure falls when upright owing to impaired release of norepinephrine, leading to dizziness. Ampreloxetine, a selective norepinephrine reuptake inhibitor, increases circulating norepinephrine levels. This study explored the safety of ampreloxetine and its effect on blood pressure and symptoms in patients with neurogenic orthostatic hypotension.

**Methods:**

A multicenter ascending-dose trial (range 1–20 mg, Part A) was followed by a 1 day, double-blind, randomized, placebo-controlled study (median dose 15 mg, Part B). Eligible patients then enrolled in a 20-week, open-label, steady-state extension phase (median dose 10 mg, Part C) followed by a 4-week withdrawal. Assessments included the Orthostatic Hypotension Symptom Assessment Scale (item 1), supine/seated/standing blood pressure, and safety.

**Results:**

Thirty-four patients (age 66 ± 8 years, 22 men) were enrolled. Part A: The proportion of participants with a positive response (i.e., increase from baseline in seated systolic blood pressure of ≥ 10 mmHg) was greater with the 5 and 10 mg ampreloxetine doses than with placebo or other active ampreloxetine doses. Part B: Seated blood pressure increased 15.7 mmHg 4 h after ampreloxetine and decreased 14.2 mmHg after placebo [least squares mean difference (95% CI) 29.9 mmHg (7.6–52.3); *P* = 0.0112]. Part C: Symptoms of dizziness/lightheadedness improved 3.1 ± 3.0 points from baseline and standing systolic blood pressure increased 11 ± 12 mmHg. After 4 weeks of withdrawal, symptoms returned to pretreatment levels. The effect of ampreloxetine on supine blood pressure was minimal throughout treatment duration.

**Conclusion:**

Ampreloxetine was well tolerated and improved orthostatic symptoms and seated/standing blood pressure with little change in supine blood pressure.

**Trial registration:**

NCT02705755 (first posted March 10, 2016).

## Introduction

Orthostatic hypotension (OH) is defined as a fall in systolic blood pressure (BP) of at least 20 mmHg or a reduction in diastolic BP of at least 10 mmHg within 3 min of standing or head-up tilt [1]. Estimates suggest that 20% of patients who meet criteria for OH have neurogenic OH (nOH) due to neurodegenerative lesions affecting the efferent sympathetic neuronal pathway, blunted reflex norepinephrine (NE) release, and insufficient vasoconstriction on standing [1–3]. In severe cases, patients with nOH can develop symptoms of cerebral hypoperfusion, generalized weakness, and an overwhelming urge to sit down [4]. This can lead to disability, poor quality of life, syncope, and falls [5, 6].

nOH is common in patients with synucleinopathies—a group of overlapping neurodegenerative diseases with intracellular deposits of misfolded α-synuclein and cell loss at various levels within the sympathetic neuronal pathway [2, 7–9]. In Parkinson’s disease (PD) and pure autonomic failure (PAF), α-synuclein aggregates as Lewy bodies and neurites mostly in the *peripheral,* postganglionic, sympathetic neurons innervating the blood vessels, and the heart [2, 7, 9]. In multiple system atrophy (MSA), the autonomic neuronal pathology occurs with a more *central* pattern. Initial loss impacts oligodendroglial cells leading to death of the sympathetic, preganglionic neurons of the spinal cord and brainstem nuclei [9]. Regardless of the site of the lesion, these three phenotypes share the failure to appropriately release NE on standing and all may lead to severe symptomatic nOH [2, 10].

Available treatment options include volume expansion with fludrocortisone or short-acting pressor agents, such as the direct alpha-1 adrenergic agonist midodrine or the norepinephrine precursor droxidopa [10–12]. However, these strategies do not target the residual sympathetic nerve activity, require multiple daily dosing, have significant side effects, and can induce supine hypertension. Moreover, many patients do not adequately respond and remain severely disabled [6, 13–15].

Norepinephrine transporter (NET) inhibitors are a novel pharmacological approach being explored as a treatment for nOH in the autonomic synucleinopathies [16]. By blocking the reuptake of NE, they harness residual peripheral sympathetic vasoconstrictor tone by prolonging the effect of released NE from the remaining postganglionic sympathetic neurons [17]. Because the central sympatholytic mechanism of NET inhibitors is not seen in patients with synucleinopathies [18], blockade of the reuptake of NE in the postganglionic sympathetic neurons may increase BP and improve symptoms [16, 17, 19].

Ampreloxetine is a novel, investigational, long-acting NE reuptake inhibitor (NRI). In addition to ongoing randomized and well-controlled phase 3 placebo-controlled studies in symptomatic nOH, ampreloxetine has previously been evaluated in up to 499 individuals, including healthy volunteers and individuals with fibromyalgia or attention deficit hyperactivity disorder. Ampreloxetine was found to be generally safe and well tolerated across these populations. Pharmacokinetic (PK)/pharmacodynamic evaluations conducted in these individuals have shown that ampreloxetine has a half-life of 30–40 h and a preferential selectivity for the NET over the serotonin transporter, with > 75% NET occupancy at a dose of 10 mg QD [20]. A phase 2 trial to investigate the safety, exploratory efficacy, and durability of once-daily oral ampreloxetine in patients with nOH due to synucleinopathies was conducted. Ampreloxetine PK results from this study have been published [21].

## Methods

### Trial oversight and registration

The phase 2 trial was a three-part, multicenter study conducted at six sites in the US (Long Beach, CA; Farmington Hills, MI; Berlin, NJ; New York, NY; Nashville, TN; Dallas, TX). The studies were conducted between September 2017 and November 2018 in accordance with the Declaration of Helsinki. Local institutional review board approval was obtained, and all participants signed informed consent. All authors reviewed the data, which was analyzed by the sponsor, and the study was funded by Theravance Biopharma R&D, Inc. The study was listed on ClinicalTrials.gov (NCT02705755) and is reported here following the CONSORT statement for reporting of clinical trials.

### Trial design

**Fig. 1:**
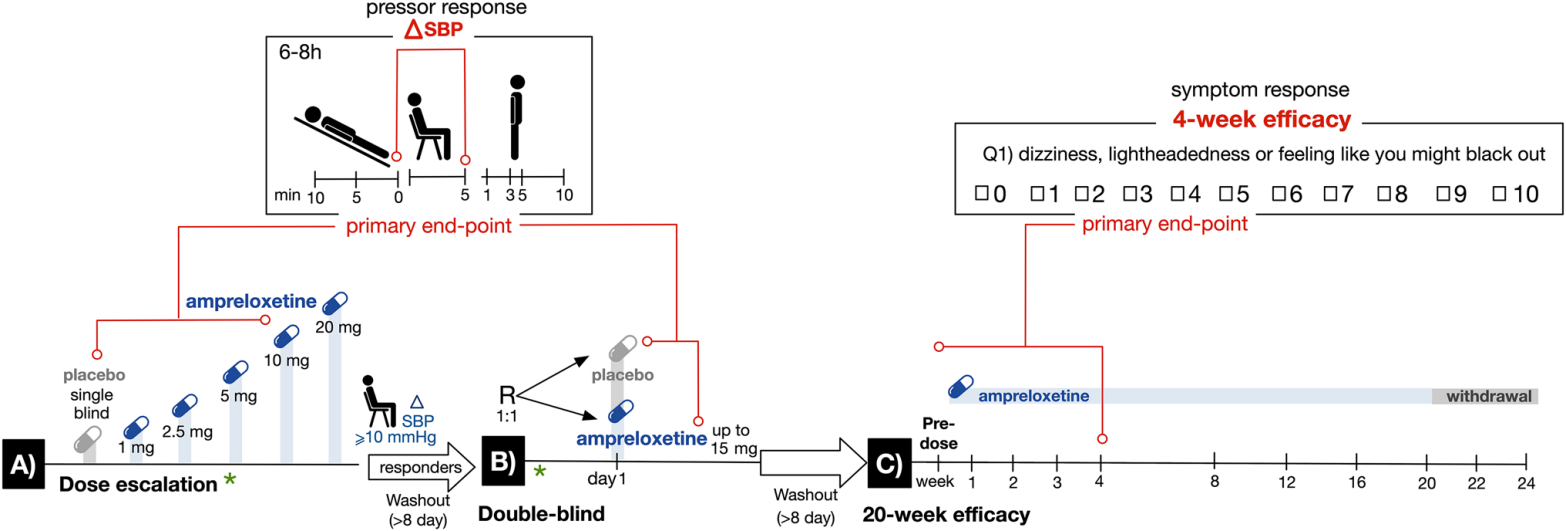
Overall trial design. Part A: Day 1, placebo was administered single blind and on subsequent days, participants received ascending doses of ampreloxetine (1–10 mg or 2.5–20 mg from day 2 to day 5). Stopping criteria included safety concerns/adverse events or seated BP > 180/110 mmHg. Responders were defined as those with a seated systolic pressor response ≥ 10 mmHg relative to placebo and underwent a washout. Part B: Participants were randomized (1:1) to ampreloxetine or placebo for 1 day. Part A and Part B were inpatient studies and the primary endpoint in both was the change in seated BP 6–8 h post-ampreloxetine. Part C: Predose assessments were taken as baseline. Participants received open-label ampreloxetine for 20 weeks as outpatients (median dose 10 mg/day). The primary endpoint in Part C was a change from baseline in OHSA #1 scores at week 4. At the end of the 20 weeks, ampreloxetine was withdrawn to determine whether symptoms diminished to baseline levels. Key: Blue pills = ampreloxetine. Gray pills = placebo. Red = primary endpoints for Parts A, B, and C. Green asterisk = main protocol amendments (i) to start the dose escalation at 2.5 mg and (ii) to eliminate Part B in order for patients to directly enter Part C. *BP* blood pressure, *h* hours, *mg* milligrams, *min* minutes, *mmHg* millimeters of mercury, *OHSA #1* dizziness/lightheadedness score on Orthostatic Hypotension Symptom Assessment (question 1), *R* randomization, *SBP* systolic blood pressure

The trial (Fig. 1) began with a single-blind, 5-day, ascending-dose inpatient study (Part A). On day 1, placebo was administered, and assessments were performed by the unblinded investigator, but the participant was blinded. On days 2–5, participants were administered ascending oral doses of ampreloxetine (dried powder mixed with filtered apple juice prepared by an unblinded pharmacist) beginning at 1 mg, followed by 2.5, 5, and 10 mg or 2.5, 5, 10, and 20 mg, always at 8 a.m. after an overnight fast. Stopping criteria included safety concerns, intolerable side effects, seated systolic BP > 180 mmHg, or seated diastolic BP > 110 mmHg. Participants were discharged and underwent washout for a minimum of 8 days. Responders (i.e., patients with an increase of ≥ 10 mmHg in seated systolic BP relative to placebo) were eligible to participate in a double-blind, placebo-controlled, randomized study (Part B). Participants were randomly assigned 1:1 to ampreloxetine or placebo using a centralized computer-generated block randomization schedule. Based on pharmacokinetic modeling of ampreloxetine accumulation after escalating doses, participants received either 1.5 times their highest effective tolerated ampreloxetine dose (dried powder mixed with filtered apple juice prepared by an unblinded pharmacist) or matching placebo for 1 day with both the investigator and the participant blinded to treatment assignment. While inpatient, ampreloxetine was administered in the morning, and participants were given a standardized low-carbohydrate meal to avoid the confounding effects of postprandial hypotension and instructed to maintain a stable fluid intake to avoid volume shifts. The study was originally designed to only include Part A and B but was modified to include Part C (an open-label extension study) after several patients who had participated in Part A and/or Part B requested compassionate-use access to ampreloxetine based on their subjective experience of reduced nOH symptoms. As a result, the protocol was amended to terminate Part B enrollment early and to initiate the Part C open-label extension phase of the study. Participants in Part C underwent a washout for a minimum of 8 days after Part A or B before enrolling in Part C. To test durability, participants were readmitted to the hospital for 4 days to repeat pretreatment evaluations and restart ampreloxetine. The dose administered in Part C (day 1) was equal to 50% of the highest tolerated dose administered in Part A. The investigators could, however, double the dose at their discretion (up to day 29) or thereafter if the sponsor agreed. They were then discharged and instructed to take ampreloxetine capsules orally in the morning before breakfast for 20 weeks, which was then followed by a 4-week withdrawal phase to determine whether their symptoms worsened and returned to baseline. In the open-label, outpatient phase, ampreloxetine could be increased to a maximum of 20 mg once daily up to the end of week 4 with at least 7 days between dose increases. If the participant developed supine hypertension or other adverse events (AEs) ampreloxetine was stopped for 3 days and restarted at 50% of the previous dose. Amendments to the protocol included dropping the 1 mg dose in Part A to begin the dose escalation at 2.5 mg and early termination of Part B allowing for patients to directly enroll in the open-label extension phase (Part C).

### Patient cohort

Eligible patients were men or women ≥ 40 years of age with a diagnosis of PD, MSA, or PAF according to established consensus criteria [1, 8, 22]. Patients were mostly recruited from those followed in specialist autonomic clinics. Non-neurogenic causes for OH, including cardiac insufficiency or overuse of anti-hypertensive drugs, were ruled out following standard-of-care clinical guidelines. The diagnosis of nOH due to efferent autonomic failure was confirmed as appropriate on autonomic testing by (1) a fall in systolic BP > 30 mmHg within 5 min of tilt, (2) lack of phase IV BP overshoot after release of the Valsalva strain, and (3) reduced respiratory sinus arrhythmia in response to deep paced breathing [2, 23]. Other short-acting pressor agents were discontinued for ≥ 5 half-lives before first study dosing on day 1. Fludrocortisone could be continued at ≤ 0.1 mg/day. Long-acting antihypertensives were not allowed. Existing treatment with short-acting antihypertensives at bedtime for supine hypertension was permitted at the discretion of the investigator. Key exclusion criteria included systemic illnesses known to produce autonomic neuropathies such as diabetes, amyloidosis, and autoimmune conditions; known intolerance to NRIs; and use of any monoamine oxidase inhibitor within 14 days of first study dosing on day 1.

### Assessments

Figure 1 shows the timing of all in- and outpatient assessments. BP was measured in the nondominant arm at heart level with an automated (or manual) sphygmomanometer after 5 and 10 min semi-supine (elevated 30°); after 5 and 10 min seated; and after 1, 3, 5, and 10 min standing immobile. In the dose-escalation trial, measurements were obtained predose and at 4, 7, 9, and 12 h postdose. In the double-blind treatment phase, measurements were obtained throughout the day. In the open-label extension phase, BP measurements were performed predose and at 4 h postdose.

Symptom burden was evaluated using the validated Orthostatic Hypotension Questionnaire (OHQ) [24]. This included the six-item OH symptom assessment (OHSA) to capture the severity of cardinal symptoms of nOH on standing (e.g., dizziness/lightheadedness/near fainting, visual problems, weakness, fatigue, trouble concentrating, and head/neck discomfort) and the four-item Orthostatic Hypotension Daily Activities Scale (OHDAS) to capture interference of low BP symptoms on activities that require standing or walking. Participants were required to rate their symptoms on a Likert scale from 0 to 10, with 10 being most severe. Additional exploratory efficacy measures in the open-label extension phase included Patient Global Impression of Severity (PGI-S) [25] assessed on an 8-point Likert scale (from 0 = not assessed to 7 = very much worse).

Safety monitoring included AE reporting [including serious AEs (SAEs)], monitoring of vital signs (including supine BP), 12-lead electrocardiogram, clinical laboratory tests (hematology, serum chemistry, and urinalysis), use of concomitant medications, and physical examination.

### Endpoints

The primary pharmacodynamic endpoint in the dose-escalation (Part A) and double-blind, placebo-controlled (Part B) inpatient studies was the change in seated systolic BP 6–8 h after drug administration. The primary pharmacodynamic endpoint in the open-label extension phase was an improvement from baseline in dizziness/lightheadedness scores (OHSA item 1) at the end of week 4 [24]. Other exploratory secondary endpoints included (1) change in the overall OHSA score, (2) change in the overall OHDAS score, (3) a change in the combined composite OHQ score, (4) change in standing systolic BP, (5) change in PGI-S, (6) duration of standing time, and (7) area under the curve for seated systolic BP 0–12 h after study drug. The pharmacokinetics and pharmacodynamics findings are reported elsewhere [21].

### Statistical analysis

Planned enrollment in Part A was 40 participants. Planned enrollment in Part B was 20 participants (10 in the ampreloxetine treatment group and 10 in the placebo group). Planned enrollment for Part C was 20 participants. If 20 participants completed week 4, 13 responders (increase in OHSA item 1 score > 2) were needed to reject the null hypothesis of response rate < 40% (power 60.1%).

BP responders were defined as those with a ≥ 10 mmHg increase in seated systolic BP after ampreloxetine administration. Participants with OHSA item 1 score of > 4 at baseline were defined as symptomatic. Improvement of 1 point (− 1) on OHSA item 1 was applied as the minimal clinically important difference based on efficacy data from the literature (MCID) [11]. Composite OHQ scores were calculated as the sum of item scores divided by number of items, as described elsewhere [24]. PGI-S reported by participants was grouped into “improvement” or “no change/worsening” in the severity of nOH symptoms compared to baseline and summarized as participant counts and percentages [26]. In the dose-escalation study (Part A), paired *t* tests were used to compare time-matched differences between each ampreloxetine dose and placebo (day 1). In the 1 day, double-blind, placebo-controlled study (Part B), the difference in seated and standing systolic BP between ampreloxetine and placebo at 4–8 h after study drug administration was determined using a mixed model for repeated measures, with treatment group, time point, and interaction between the treatment group and time point as fixed factors. In the open-label extension phase (Part C), baselines for BP, OHSA, OHDAS, and PGI-S were determined after readmission before the first dose of ampreloxetine. All analyses in Part C were descriptive. No imputation for missing data was performed. Safety data for all studies were listed by participant and summarized using participant count and percentage. Participants with severe symptoms (defined as OHSA item 1 score > 4 points at baseline) were identified as a subgroup for additional analyses.

There was no formal hypothesis testing, and all results including *P* values were descriptive. Blood pressure data during Part B were tested for significance using Student’s *t* test, with the empirical assumption that the data were not normally distributed given the small number of subjects. Analysis was performed with SAS version 9.3 or later. Descriptive statistics by treatment are reported as mean ± SD; comparisons with placebo are reported as mean ± SEM unless otherwise stated.

## Results

### Patient characteristics and study flow

A total of 34 patients were enrolled, and 33 received at least one dose of ampreloxetine. The demographics are listed in Table 1 and reflected enrollment at specialized autonomic centers [23]. There was a predominance of white men. Mean age was 66 (range 51–83) years. Approximately 50% of participants met diagnostic criteria for MSA. Twenty-six out of 34 participants were unable to remain standing for 10 min (Table 1). The orthostatic heart rate rise was significantly impaired and fell within the diagnostic range for nOH [27]. Twenty-one participants were enrolled in the open-label extension phase (Part C), with 17 completing assessments at week 4, 12 completing week 20, and 11 completing week 24. Seventeen participants entering the open-label extension phase were determined to be highly symptomatic at baseline with an OHSA item 1 score > 4; of these, 13 completed week 4, 8 completed week 20, and 7 completed week 24.

**Table 1:** Demographics and patient characteristics

	Dose escalation (Part A)	Double blind (Part B)		20-Week open-label extension (Part C)	
		Ampreloxetine	Placebo	All	Symptomatic^a^
Demographics					
*n*	34	5	5	21	17
Mean age, years	66 ± 8	66 ± 5	65 ± 9	64 ± 8	65 ± 8
Male:female, *n* (%)	22:12 (65:35)	3:2 (60:40)	3:2 (60:40)	12:9 (57:43)	8:9 (47:53)
Race, white, *n* (%)	31 (91)	5 (100)	4 (80)	18 (86)	15 (88)
Diagnosis					
MSA, *n* (%)	18 (53)	2 (40)	3 (60)	12 (57)	9 (53)
PD, *n* (%)	9 (27)	2 (40)	1 (20)	5 (24)	4 (24)
PAF, *n* (%)	7 (21)	1 (20)	1 (20)	4 (19)	4 (24)
Symptoms					
OHSA #1 > 4, *n* (%)				17 (81)	17 (100)
OHSA #1 (points)	5.9 ± 3.28	6.2 ± 1.92	6.8 ± 3.49	6.6 ± 3.12	7.8 ± 1.74
OHSA composite (points)	–	–	–	4.30 ± 2.623	5.18 ± 2.067
OHDAS composite (points)	–	–	–	6.72 ± 2.726	7.53 ± 2.128
OHSA #1 (range)	0–10	4–9	1–10	0–10	5–10
Standing duration < 10 min	26 (76)	5 (100)	3 (60)		
Systolic BP, mmHg					
Supine	135 ± 26	134 ± 24	121 ± 22	130 ± 24	132 ± 24
Seated	115 ± 24	105 ± 33	95 ± 21	107 ± 25	107 ± 26
3 min standing	84 ± 20	83 ± 30	75 ± 8	84 ± 18	83 ± 17
Lowest standing	78 ± 17	81 ± 19	67 ± 9	74 ± 17	75 ± 18
Heart rate, bpm					
Supine	67 ± 9	61 ± 2	70 ± 9	68 ± 7	68 ± 7
Seated	74 ± 10	69 ± 7	70 ± 7	76 ± 9	77 ± 10
3 min standing	89 ± 20	76 ± 10	81 ± 3	84 ± 10	85 ± 11
ΔHR:ΔSBP (ratio)	0.38 ± 0.249	0.31 ± 0.076	0.28 ± 0.302	0.47 ± 0.408	0.49 ± 0.437
Supine NE (pg/ml)^b^				310 ± 211	301 ± 231

Data are mean ± SD or *n* (%). For Parts A and B, standing duration < 10 min captures *n* (%) of patients who were unable to stand for 10 min owing to symptoms consistent with cerebral hypoperfusion

*BP* blood pressure, *bpm* beats per minute, *ΔHR:ΔSBP (ratio)* index of baroreflex function calculated by change in HR divided by change in systolic BP at 3 min of standing (ratio < 0.495 bpm/mmHg indicates neurogenic OH) [27], *HR* heart rate, *min* minutes, *mmHg* millimeters of mercury, *MSA* multiple system atrophy, *NE* norepinephrine, *OHDAS* Orthostatic Hypotension Daily Activities Scale, *OHSA #1*  dizziness/lightheadedness score on Orthostatic Hypotension Symptom Assessment (question 1), *PAF* pure autonomic failure, *PD* Parkinson disease, *pg* picogram, *SD* standard deviation, *year* years

^a^In Part C, the symptomatic subgroup presented comprises patients with an OHSA #1 > 4 points

^b^For additional information on norepinephrine levels and pharmacodynamic studies, see Lo A et al. *Clin Auton Res*. 2021. 31(3):395–403 [21]

**Fig. 2:**
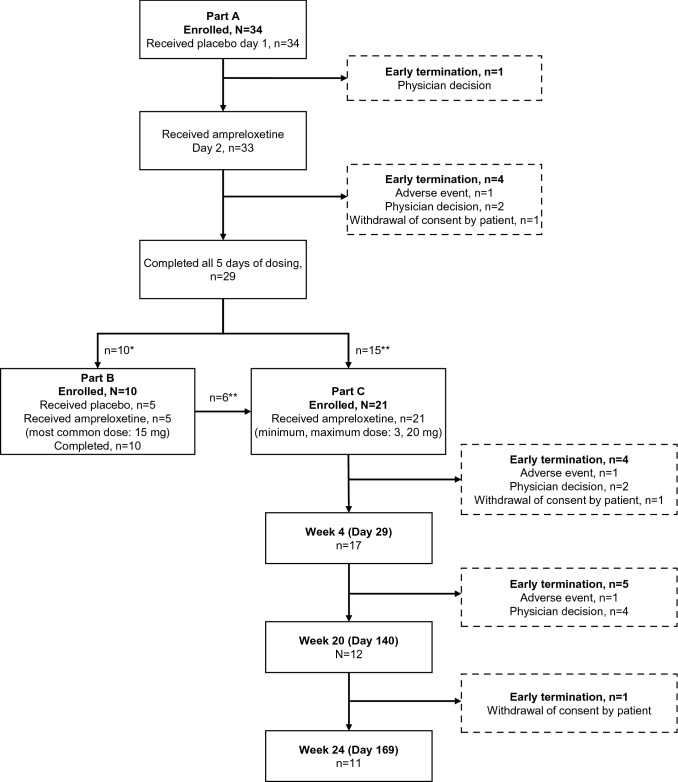
Consort diagram describing the flow of participants through the study. *Participants who completed Part A and entered Part B before amendment. **Number of participants who entered Part C after completion of Part B (before amendment) and those who directly entered Part C from Part A (after amendment). *mg* milligrams, *n* number

Reasons for dropouts in the dose-escalation study (Part A) were physician decision (*n* = 3), an AE (*n* = 1), and withdrawal of consent (*n* = 1). No participant withdrew in the double-blind, placebo-controlled study (Part B). In the open-label extension phase (Part C), seven discontinuations were in participants with MSA, three of whom discontinued owing to physician decision, which most likely reflects the rapid progression of this disorder, and two discontinuations were owing to unrelated AEs and two were withdrawal by participant. Three non-MSA participants discontinued the study owing to physician decision. Five participants temporarily interrupted and then restarted ampreloxetine in Part C. Figure 2 describes the participant flow and reasons for dropouts.

### Prior and concomitant medications

At enrollment (Part A), 24/34 (71%) participants had received any prior pressor medication. The most common prior pressor medications were midodrine (*n* = 17) and droxidopa (*n* = 8), which were stopped prior to start of study dosing. During the study, 17/34 (50%) received concomitant fludrocortisone. Three patients with MSA were receiving pharmacological treatment for supine hypertension (2 patients were treated with a 0.1 mg/h nitroglycerine transdermal patch at bedtime and 1 patient was treated with losartan).

### Dose-escalation study (Part A)

**Fig. 3:**
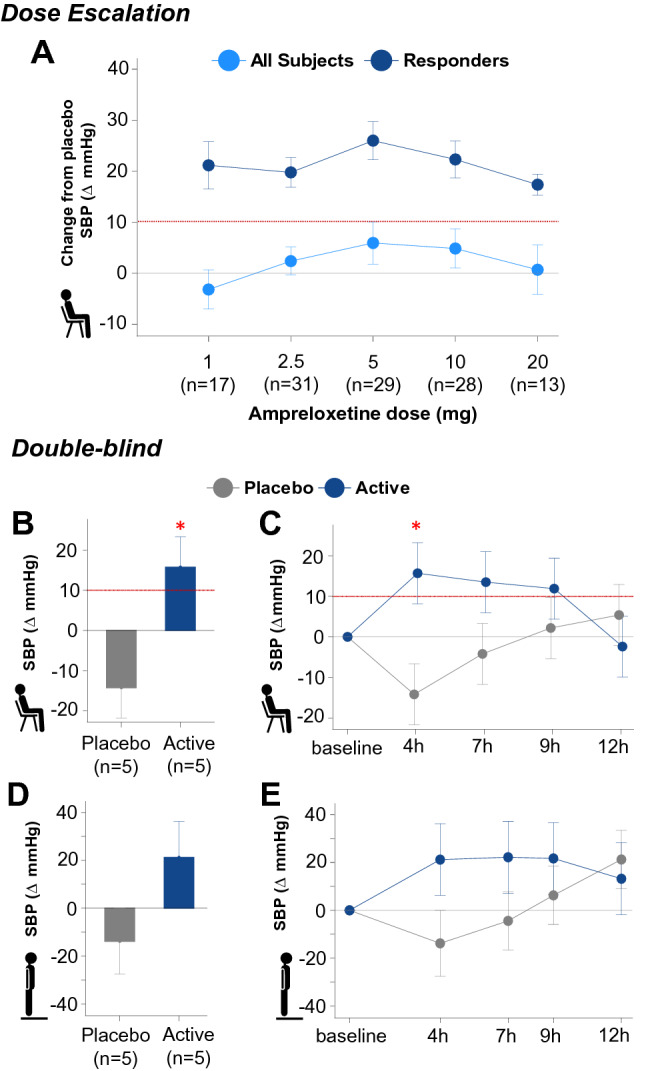
Pressor response to ampreloxetine.** a** Mean ± SE placebo time-matched change in seated SBP (primary endpoint) throughout dose escalation in the overall cohort (light blue) and responders (dark blue). Owing to an early trial amendment, not all participants received the 1 mg dose on day 1. Responders were not escalated to a higher dose. **b** Mean ± SE change in seated SBP 4 h after ampreloxetine (15 mg, *n* = 4, 10 mg, *n* = 1) vs. placebo (*n* = 5) in the 1 day double-blind, placebo-controlled randomized study. **c** Timeline of change in seated BP over 12 h after ampreloxetine and placebo. **d** Change in standing SBP 4 h after ampreloxetine and placebo. **e** Timeline of changes in standing SBP when assigned to ampreloxetine vs. placebo. Red dotted line denotes cutoff for a pressor responder, determined by a change in seated SBP ≥ 10 mmHg compared to baseline. In panels C through E, data shown as least squares mean ± SE; blue represents ampreloxetine, gray represents placebo. Red star indicates significance. *BP* blood pressure, *mmHg* millimeters of mercury, *SBP* systolic blood pressure, *SE* standard error

Seventeen participants received 1 mg, 31 participants 2.5 mg, 29 participants 5 mg, 28 participants 10 mg, and 13 participants 20 mg (Part A). Figure 3a shows the increase in seated systolic BP relative to placebo at each dose. The percentage of responders (defined as an increase in seated systolic BP of ≥ 10 mmHg relative to placebo) at 6–8 h after study drug administration was highest at the 5 mg (43%) and 10 mg (39%) ampreloxetine doses. Responders did not escalate to higher doses. Of the 13 participants receiving the 20 mg maximal dose, no additional benefit to seated systolic BP was observed (Fig. 3a).

### Double-blind, placebo-controlled study (Part B)

Ten participants were enrolled in the double-blind, placebo-controlled study (Part B). In the ampreloxetine treatment group, the most common dose was 15 mg (4 participants received 15 mg, 1 participant received 10 mg). As shown in Fig. 3b, 4 h after ampreloxetine, seated systolic BP increased by 15.7 mmHg compared to a decrease of 14.2 mmHg after placebo, yielding a least square mean difference of 29.9 mmHg (95% CI 7.6–52.3; *P* = 0.0112). An increase in standing BP was also observed with ampreloxetine (Fig. 3d). Four hours after ampreloxetine, standing systolic BP at minute 3 was numerically higher (35.0 ± 20.6 mmHg) than placebo (95% CI − 18.8 to 88.8). The pressor response subsided after 8 h and within 12 h was not different from placebo (Fig. 3c and e). In total, 4/5 of the patients receiving ampreloxetine reported a ≥ 1-point improvement in their OHSA item 1 score vs. 2/5 of the participants receiving placebo.

**Fig. 4:**
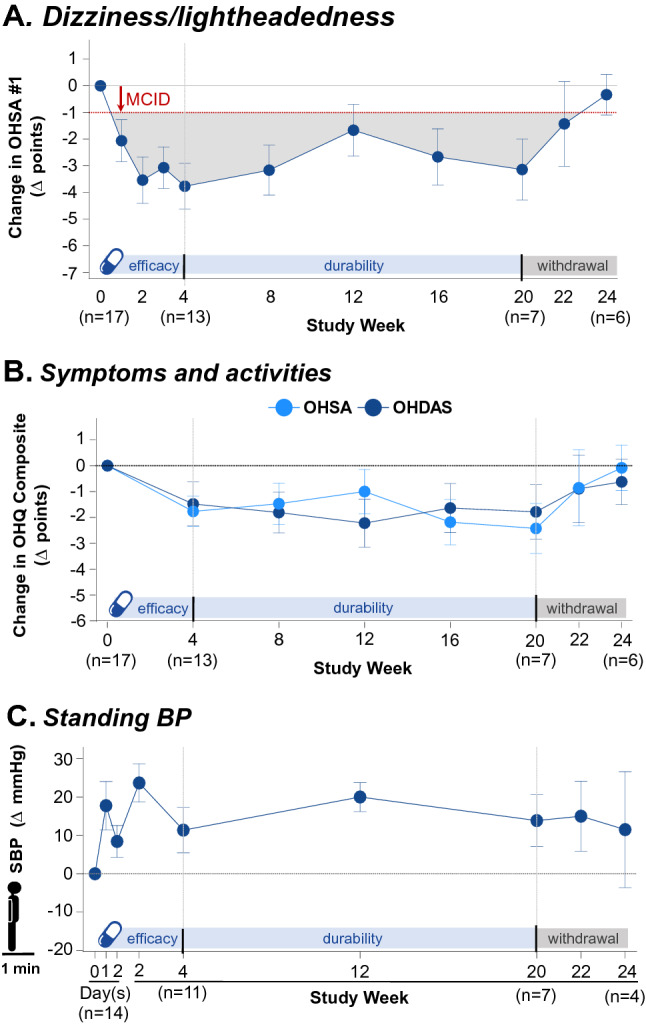
Symptoms and BP response over 20 weeks of open-label extension. **a** Mean change from baseline in dizziness/lightheadedness scores (OHQ symptom assessment item 1) in the subset of symptomatic participants (OHSA#1 > 4 points). A decrease in scores indicates clinical improvement. The primary endpoint was the change observed at week 4. Values below dotted red line indicate improvement above the MCID. The observed effect was sustained throughout the 20 weeks. After withdrawal of ampreloxetine, symptoms diminished to baseline levels. **b** Similar pattern of changes observed with the composite scores of symptom severity (OHSA) and impact of symptoms on activities of daily living (OHDAS). **c** Change from baseline in systolic BP at 1 min standing over the 20-week trial. Note, after withdrawal of ampreloxetine, most patients (*N* = 7) resumed taking other pressor agents. An increase in BP was observed off-treatment without corresponding improvement in symptoms. Data are mean ± standard error. *BP* blood pressure, *MCID* minimal clinically important difference, *OHDAS* Orthostatic Hypotension Daily Activities Scale, *OHQ* Orthostatic Hypotension Questionnaire, *OHSA* Orthostatic Hypotension symptom assessment, *OHSA #1*  dizziness/lightheadedness score on Orthostatic Hypotension Symptom Assessment (question 1)

### Symptom improvement on open-label extension

A total of 21 patients were enrolled in the open-label extension (Part C). The median dose of ampreloxetine in the 20-week treatment phase was 10 mg. As shown in Fig. 4a, during the entire treatment duration of 20 weeks, average improvement in OHSA item 1 in the symptomatic subset was consistently > 1 point (MCID). Symptoms of dizziness/lightheadedness improved as early as week 1. Analysis of symptomatic patients (OHSA item 1 score > 4 at baseline) revealed a 3.8 ± 3.1 point (mean ± SD) decrease in dizziness/lightheadedness scores at the end of 4 weeks of treatment. On treatment, 77% of symptomatic participants reported ≥ 2-point improvement, 69% reported ≥ 3-point improvement, and 54% reported ≥ 4-point improvement at week 4. Symptomatic improvement was sustained at the end of week 20 with a mean decrease in symptom scores of − 3.1 ± 3.0 points (mean ± SD); 86% reported ≥ 1-point improvement, 71% reported ≥ 2-point improvement, and 43% of patients reported ≥ 4-point improvement. After ampreloxetine withdrawal, symptoms worsened and returned to pretreatment levels despite participants restarting alternative pressor agents. After 4 weeks of ampreloxetine withdrawal, mean (± SD) improvement had dropped to − 0.3 ± 1.9 points, and the proportion of participants reporting ≥ 1-point improvement dropped to 50%; no patient reported improvement of > 2 points. As expected, the nonsymptomatic subset also reported improvement, but the improvement was less pronounced than that seen with the symptomatic subset of participants.

Similarly, improvements in the OHSA and OHDAS composite scores were seen as early as week 1, were sustained throughout the 20 week treatment period, and diminished to pretreatment levels after ampreloxetine withdrawal (Fig. 4b).

### Durability of the pressor response on open-label extension

**Fig. 5:**
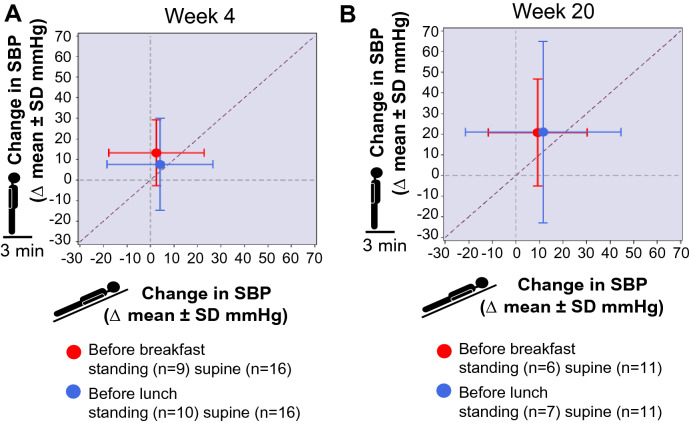
Responses in autonomic failure. **a** Change in SBP supine vs. change in 3-min standing SBP at week 4. Data are mean ± SD. A 5-mmHg increase in supine SBP was accompanied by an approximately 10-mmHg increase in standing SBP. **b** The durability of the pressor effect supine vs. standing was demonstrated to a greater degree on 3-min standing SBP at week 20. The increase in standing SBP exceeded the increase in supine SBP in participants receiving ampreloxetine. Red indicates before breakfast; blue indicates before lunch. Data from Part C; open-label extension. *mmHg* millimeters of mercury, *pg* picogram, *SBP* systolic blood pressure, *SD* standard deviation

Throughout the 20 weeks of treatment with ampreloxetine, standing systolic BP was increased from baseline. As shown in Fig. 4c, the pressor response was similar at week 4 (9.0 ± 23.6 mmHg) and week 20 (10.8 ± 12.1 mmHg). In the withdrawal phase, 7/11 (64%) patients resumed treatment with their other pressor agents (midodrine and droxidopa). Ampreloxetine withdrawal reversed the symptomatic benefit, with dizziness/lightheadedness scores (OHSA item 1) returning to baseline over the 4 weeks (Fig. 4a). The improvement in activities of daily living was lost after withdrawing ampreloxetine, despite most participants restarting other pressor agents (Fig. 4b). In the subset of participants who were symptomatic at baseline, average predose standing time was 5.3 ± 4.1 min (*n* = 17). In the open-label study, regression analysis of the pressor effect supine vs. standing showed a greater effect on BP in the upright position at week 4 and week 20 (Fig. 5a and b). At the end of the 20-week, open-label extension phase, standing time increased by 4 min, and participants were able to remain standing for an average of 8.6 ± 5.9 min (*n* = 7). After ampreloxetine withdrawal, the improvement in standing time was lost, and standing duration returned to baseline levels (4.8 ± 4.4 min, *n* = 6). In the 4 week withdrawal phase, only two participants were able to stand for 3 min to complete the BP measurement. At the end of the 4 weeks of withdrawal, symptoms had worsened ≥ 5 points in 80% of the participants who answered the global impression assessment scale (*n* = 8/10).

### Safety profile

Table 2 provides a full listing of AEs. Ampreloxetine was generally well tolerated. In the dose-escalation study (Part A), there were no SAEs. The most common AEs were headache (5.9%), urinary tract infection (2.9%), and constipation (5.9%); only 1 AE led to study drug discontinuation. In Part B, the only AE reported in a patient randomized to ampreloxetine was a urinary tract infection in a participant with MSA, which was considered to be unrelated to study drug. In the open-label extension phase (Part C), 18 of 21 participants reported at least 1 AE; the majority (61.9%) were moderate or severe. In Part C, five patients (23.8%) experienced at least one SAE, and none of the SAEs were considered related to study drug. No SAEs were of a psychiatric or cognitive nature. AEs led to permanent study discontinuation for two (9.5%) participants in Part C. The most common AEs were urinary tract infection (23.8%), hypertension (19.0%), and headache (14.3%) and were considered not related to ampreloxetine. No deaths were reported in this study. Overall, the most TEAEs considered to be study treatment related were headache in four participants (2 each in Part A and Part C) and hypertension in two participants (Part C).

**Table 2:** Treatment-emergent AEs

*n* (%)	Part A						Part B		Part C
	Ampreloxetine								
	Placebo (*n* = 34)	1 mg (*n* = 17)	2.5 mg (*n* = 31)	5 mg (*n* = 29)	10 mg (*n* = 28)	20 mg (*n* = 13)	Placebo (*n* = 5)	Ampreloxetine (*n* = 5)	Ampreloxetine (*n* = 21)
All	7 (20.6)	4 (23.5)	5 (16.1)	3 (10.3)	6 (21.4)	2 (15.4)	0	1 (20.0)	18 (85.7)
AEs related to drug	0	1 (5.9)	2 (6.5)	2 (6.9)	4 (14.3)	2 (15.4)	0	0	8 (38.1)
Serious AEs	0	0	0	0	0	0	0	0	5 (23.8)
Serious AEs related to drug	0	0	0	0	0	0	0	0	0
AEs leading to permanent discontinuation of drug	0	0	1 (3.2)	0	0	0	0	0	2 (9.5)
Deaths	0	0	0	0	0	0	0	0	0
≥ 2 AEs across all parts of the study by MedDRA preferred term									
Headache	2 (5.9)	0	1 (3.2)	1 (3.4)	1 (3.6)	1 (7.7)	0	0	3 (14.3)
Urinary tract infection	1 (2.9)	0	0	1 (3.4)	1 (3.6)	0	0	1 (20.0)	5 (23.8)
Hypertension	0	0	0	0	0	0	0	0	4 (19.0)
Nausea	1 (2.9)	0	0	0	0	0	0	0	2 (9.5)
Chest discomfort	0	0	0	0	0	0	0	0	2 (9.5)
Constipation	2 (5.9)	0	0	0	0	0	0	0	0
Dizziness	0	0	0	0	0	0	0	0	2 (9.5)
Ear pain	0	0	0	0	1 (3.6)	0	0	0	1 (4.8)
Hematuria	0	0	0	0	0	0	0	0	2 (9.5)
Laceration	0	0	0	0	0	0	0	0	2 (9.5)
Loss of consciousness	1 (2.9)	0	0	0	0	0	0	0	1 (4.8)
Musculoskeletal pain	0	0	0	0	0	0	0	0	2 (9.5)
Orthostatic hypotension	0	0	0	0	1 (3.6)	0	0	0	1 (4.8)
Syncope	0	0	0	0	0	0	0	0	2 (9.5)

*AE* adverse event, *MedDRA* Medical Dictionary for Regulatory Activities, *mg* milligrams

## Discussion

A pressor effect was observed with ampreloxetine treatment in the dose-titration phase, as evidenced by approximately 40% of participants experiencing a ≥ 10 mmHg increase in seated systolic BP relative to placebo. Moreover, in the double-blind study, BP was significantly higher on ampreloxetine compared to placebo. There was durable improvement in symptoms over 20 weeks of open-label treatment, which worsened back to baseline levels once ampreloxetine was withdrawn.

These findings are encouraging. To date, there are no available therapies for nOH in patients with autonomic synucleinopathies that have shown a durable effect beyond 1 week in clinical trials. Therapies with approved available pressor agents (midodrine and droxidopa) have been shown to exacerbate supine hypertension [12, 15], which is a limiting factor in achieving therapeutic benefit [13, 28]. The mechanism of action of ampreloxetine as an NRI provides an alternative strategy to available pressor agents [17]. Plasma measurements of NE in this target population suggest that ampreloxetine blocks NE reuptake upon release, increasing its bioavailability at the neurovascular junction, and thus may harness the effect of the residual postganglionic sympathetic neurons preferentially when activated by the baroreflex on standing [21]. This is a more physiological approach that may reduce the risk of supine hypertension, as it would be most likely to potentiate sympathetic tone when activated upon standing [2]. The selectivity of ampreloxetine for the NET over the serotonin transporter and its long plasma half-life with stable plasma levels over 24 h suggest that it may be of potential benefit as a once-daily, durable treatment for nOH [20, 29]. We did not formally restrict recruitment to patients who failed treatment with other pressor agents as we wanted a representative sample of typical patients with nOH at various stages of disease.

Ampreloxetine had a favorable safety profile in participants with nOH. The most common AEs were urinary tract infections, which occur frequently in patients with autonomic synucleinopathies owing to bladder dysfunction, especially those with MSA, who made up the largest proportion of patients recruited into the trial [30]. Supine hypertension, particularly at night, is inherent to this group of disorders with or without pressor agent therapy [13, 28]. Although no signal was observed that ampreloxetine worsened supine hypertension at 4 and 20 weeks of treatment (and preferentially impacted standing BP; Fig. 5), hypertension was a reported AE in four patients (19%), but only in Part C, and this effect of ampreloxetine will continue to be investigated in future studies. Of note, data were only available for seven participants at week 20; longer-term studies with larger numbers of participants are ongoing to continue to investigate.

Ampreloxetine doses between 5 and 10 mg had the highest percentage of pressor responders relative to placebo (Fig. 3). A further increase in the proportion of BP responders was not observed at the 20 mg dose and may be due to steady-state concentrations being reached as preliminary pharmacokinetic analysis of participants in Part C showed that steady-state concentrations were achieved between days 1 and 15. These results, combined with the lack of any appreciable difference in safety or tolerability between 5 and 10 mg doses, led to the decision to proceed at a dose of 10 mg in the phase 3 studies. The pressor response to ampreloxetine and improvement in symptoms in the double-blind, placebo-controlled study (Part B), based on ten participants, provides an encouraging signal for efficacy.

The results of the open-label extension phase (Part C), based on 17 participants who were symptomatic at baseline, suggest that symptomatic benefit is achieved within 1 week and is durable for up to 20 weeks of treatment. In the withdrawal phase, despite restarting other pressor agents, symptoms worsened back to baseline after ampreloxetine was discontinued, which suggests that the symptom-related benefit may have been specific to ampreloxetine. We did not see a clear decrease in blood pressure after ampreloxetine withdrawal, probably owing to most patients (*n* = 7) restarting their previous medications for nOH. Two previous studies have shown that although midodrine and NET inhibitors both improved orthostatic BP, NET inhibition was better in terms of improving symptoms in participants with nOH [19, 31].

We did not detect a difference in clinical benefit between participants with a central preganglionic phenotype of autonomic failure (e.g., MSA), who are presumed to have sparing of postganglionic sympathetic fibers, and those with Lewy body disorders, in which the brunt of the α-synuclein deposition is thought to occur in the postganglionic fibers (i.e., PD and PAF) [7, 17, 23, 30]. This may be because the sample size was too small to detect a clinical effect by disease state. It may also be that the neuropathological phenotypes of autonomic failure are less precise or frequently mixed according to disease state. Many patients with PD and PAF have some sparing of the postganglionic sympathetic nerve fibers combined with denervation super-sensitivity within the vasculature [10]. As is observed with other pressor agents [10, 32], the levels of NE and the degree of residual sympathetic vasoconstrictor tone may determine the response to treatment rather than the diagnosis per se. In line with this assumption, we found that the pressor response to standing following ampreloxetine was related to the levels of NE (Fig. 5), which is a biomarker of overall residual sympathetic tone in patients with autonomic failure [2, 32, 33]. We will continue to explore the hypothesis that the extent of denervation drives the clinical responsiveness to NET inhibition in our larger patient cohort.

Our study has some limitations, including the small number of participants, particularly in Part B. Furthermore, the 20-week treatment (Part C) was an open-label extension with no placebo control. The prolongation of standing time after treatment with ampreloxetine and worsening after withdrawal is an encouraging signal; however, a larger cohort is needed to confirm this. Enrollment was limited to specialist autonomic clinics because we wanted to carefully observe the findings. The fall in BP 4 h after placebo observed in Part B (Fig. 3c and e) could have had a number of causes including small sample size, and because of variation in the timing of meals, it was not possible to determine whether it was caused by postprandial splanchnic vasodilatation. A larger sample size is needed to better understand whether the AEs related to hypertension are de novo, exacerbation of preexisting supine hypertension, or part of normal disease variability. At week 12 (Fig. 4c), there was not always a tight correlation between the degree of hypotension and severity of orthostatic symptoms, which could be due to several physiologic factors including cerebral autoregulation. The ampreloxetine phase 3 program includes and should allow us to examine factors that influence responsiveness to ampreloxetine in a larger sample size of patients. The dropouts seen throughout the trial likely reflect the high proportion of MSA participants enrolled in the phase 2 program and the rapid worsening of the participants’ underlying neurological disease and progressive difficulty with mobility and attending in-clinic follow-up visits. This underscores the need for a decentralized trial design as a retention strategy with remote visits for future clinical trials.

In conclusion, the results of this small phase 2 clinical trial suggest that once-daily 10 mg ampreloxetine is generally safe and well tolerated and may have a durable clinical benefit over 5 months of treatment. The data from this study informed the design and initiation of two placebo-controlled phase 3 clinical trials and an extension study to further evaluate the longer-term efficacy and safety of a once-daily 10 mg dose of ampreloxetine in patients with symptomatic nOH (NCT03750552, NCT03829657, and NCT04095793). The phase 3 program includes and should allow us to examine factors that influence responsiveness to ampreloxetine in a larger sample size of patients.

## Data Availability

Theravance Biopharma (and its affiliates) will not be sharing individual deidentified participant data or other relevant study documents.
